# Meaning in Music Framed: The Four ‘Eff’ Processes (Fit, Affiliation, Facilitation, and Fluency)

**DOI:** 10.3390/bs15040546

**Published:** 2025-04-17

**Authors:** Emery Schubert, Anthony Chmiel

**Affiliations:** 1 Empirical Musicology Laboratory, UNSW Sydney, Sydney, NSW 2033, Australia; 2 Sydney Conservatorium of Music, The University of Sydney, Camperdown, NSW 2050, Australia; anthony.chmiel@sydney.edu.au

**Keywords:** framing, music, facilitation: fluency, fit, affiliation, meaning, pleasure

## Abstract

Music can evoke powerful, positive, and meaningful experiences, but how does its potential to evoke such experiences come about? Listening to the music itself is critical, but referents (the thoughts, ideas, events, and affects associated with the music) are also relevant. We found a lack of understanding in the literature regarding the processes through which music evokes meaning through referents. To address this lacuna, we built on modern conceptions of framing theory. The following four framing processes were proposed, with each acting on different time scales (shortest [S] to longest [L]), and with an increasingly top-down [T] influence: (1) fluency [S]—the ease with which the accompanying information (about the music) can be mentally processed, with easy-to-process material leading to ‘increased preference/positive evaluation of the music’ [IPPE]; (2) facilitation—the content of the messaging directly influences IPPE, for example, when referring to the beauty of the music or the talent of the composer; (3) affiliation—when social influences imbue the music with meaning; and (4) fit [L, T]—when the other processes lead to long-term personal and cultural IPPE through norms and habits. Together, these processes can be applied to provide a comprehensive account of how musical meaning and preferences are developed. Three case studies show how these processes can be applied to the extant literature: why negatively framed music only has a relatively small (negative) impact on IPPE; why adding crowd sounds to recorded music only has a small effect; and how ‘labels’ such as Beethoven and Mozart become established and then impose top-down influence on music’s meaning.

## 1. Introduction

How meaningful music is to an individual will drive their motivation to explore and listen to music (Reybrouck et al., 2024). Music acquires its various meanings, including the evocation of meaning in the listener, from the associations formed with stimuli outside the music, as well as in terms of the music itself. Numerous researchers have presented a range of perspectives on these types of meanings and which—if any—are more important or relevant to musical meaning. Among the most influential theories in music psychology is the work by Meyer (1956), who proposed that emotion and meaning in music, at a broad level, comes about through the direct meaning that is formed from listening to the music (which he referred to as ‘absolutist’) and meanings that arise from the links (the thoughts, ideas, images, analogies, references, topics, resemblances, etc.) triggered by the music (which he termed ‘referentialist’).

Since Meyer’s work, much discussion of how meaning is formed has been discussed from historical (Chua, 1999; Paddison, 2010; Semi, 2021), philosophical (Davies, 1994; Robinson, 1997; Paddison, 2020), and psychological (Watt & Ash, 1998; Cross & Tolbert, 2009; Margulis et al., 2022) perspectives. In empirical research, our understanding of absolutist meaning formation has progressed, with a strong body of evidence that meaningful, positive connotations emerge through pure exposure to music, leading to positive affective responses such as aesthetic emotions (awe, wonder, and interest) and pleasure (Margulis, 2014; Schubert et al., 2014; Schubert et al., 2016; Chmiel & Schubert, 2017). The psychological processes that drive referentialist meaning making have not, however, progressed as far, and the present paper aims to address this lacuna.

One weakness in Meyer’s classification system (which includes absolute and referential ways of forming meaning) is that it does not explain the process by which music acquires meaning, and we feel this is a gap that has not been adequately addressed since. However, the subsequent seven decades of research has seen the development of theoretical tools that can advance our understanding. Of note in this paper is a novel application of ‘framing’ to explain how meaning comes to imbue music. We will outline the concept of framing and introduce four processes of framing, each playing a role in the formation of meaning. We will then discuss how existing research can be better understood through framing processes via three case studies. Although meaning overlaps with pleasure and powerful feelings, we note here that much of the discussion that follows refers to the pleasurable (or non-pleasurable) aspects. This is because the bulk of research investigates valence (positive or negative). However, we aim to provide a theoretical grounding that encapsulates musical meaning in a broader sense (both how the music creates meaning in the individual, and the meanings that the music represents).

## 2. Framing

Framing, as the term is used in social science and in the sub-discipline of Communication specifically, refers (usually) to linguistic tools that bias the interpretation of factual information—deliberately or incidentally. For example, describing (i.e., framing) an event as ‘catastrophic’ versus ‘overwhelming’, or stating that ‘10% of the population will be lost’ versus ‘90% of the population will be saved’ can produce consistently and distinctly different interpretations of an event (Tversky & Kahneman, 1981). Carpenter (2022, p. 2793) refers to a *Framing Effect*: “[t]he way that information is presented systematically changes perceptions of and reactions to that information, even when the details remain objectively equivalent”. As Entman (1993) states in his seminal paper on the topic, “[t]o frame is to select some aspects of a perceived reality and make them more salient…” (p. 52).

Framing has origins in the classical Greek technique of rhetoric, as an essential ingredient of political persuasion (Hogan, 2012). It is also importantly related to ‘suggestibility’, a phenomenon that “facilitate[s] or enhance[s] the probability of a suggestion being accepted and believed”, and ‘suggestion’, which “once accepted has the capacity, (like other strong beliefs) to exert profound changes on a person’s mood, thoughts, perceptions, and ultimately their behaviors” (Halligan & Oakley, 2014, abstract).

Individual differences are likely to be involved in responding to such frames. For example, Escolà-Gascón et al. (2023) found that schizotypal, paranoid, and histrionic personality types are more likely to accepting and internalising negative or fake information, suggesting that the strong negative absorption of frames occurs through individual differences, and so impacts on the averaged response when there is an uncontrolled heterogeneity of the sampled listeners. A personal connection with the frame will have an impact, too (e.g., A. N. Krause et al., 2024).

In recent years the meaning of framing has been extended to include any means available to modify the view of an idea, object, event, or situation, which in the present study refers to music, but can be applied to any aesthetic object or event (not to mention any information such as news and advertising). Because framing can occur incidentally (that is, without deliberate planning), the term can be broadened out to mean any information or artefacts accompanying an object/event (outside the object/event itself), not just verbal information, and so can include, in addition to deliberately presented verbal information, incidental verbal information, as well as a host of other signals available through environmental and social/cultural settings (e.g., Hullman & Diakopoulos, 2011).

Framing objects and events impacts the way we think about them. Cognitive processing (thought and action) is at the core of the framing effect (Entman, 1993, p. 56). Historically, the arts have been used to frame other messages, such as moral outcomes, religious rites, and political power/oppression (Helmers, 2004). In this paper, we focus on framing in the other direction—how information surrounding a work of art (with our focus on music) can influence *its* meaning (which includes its evocation of meaning). This is broadly in line with Entman’s account, given that the focus is still on cognitive processing (in this case, of music). We will argue that framing impacts meaning formation in music and provides a novel epistemology that accounts for referential meanings in music.

Building on the work of Goffman (1975), Wald-Fuhrmann et al. (2021) note four broad types of social constructions that can frame music, as follows:

A. Places (e.g., living rooms, cars, concert halls, and public areas);
B. Situations (e.g., commuting to work, a romantic dinner, a church service, being alone, or being with others);
C. Media (e.g., live, recording, and digital streaming);
D. Discursive contexts (such as a culture’s overarching art and music concepts, or the aesthetics of specific musical styles and genres), all of which are socioculturally determined (p. 3, enumeration added).

Any setting of these constructions forms the circumstance under which the music listening takes place. For example, a white female resident of San Francisco listening to a piece of music over a streaming service, in a car on the way to a romantic dinner with a long-term partner, is a special case of the circumstances framing the listening experience. Framing also shapes and organises mental processes. As Wald-Fuhrmann et al. (2021) put it, music is integrated by frames: “frames in which music is embedded suggest specific listening modes (e.g., attentive, non-attentive, analytical, and emotional), listening behaviors (e.g., sitting still vs. gesturing or dancing), or functions attributed to the music (e.g., for its own sake or aesthetic pleasure vs. mood management, atmosphere creation, or social bonding)” (p. 3). The concept of framing places focuses on variables outside the music that colour how music is experienced, namely habits, environments, and cultures in which the listener is situated.

While Wald-Fuhrmann et al. (2021) cover the broad sweep of possible ways for framing music, we were not able to identify a unifying framework describing the specific processes through which frames lay down the meaning that is associated with and triggered by music (work of art, etc.). By wedding the literature on the different ways musical meaning is acquired with framing (the cultural and psychological reasons alluded to above), we propose four specific processes through which framing can instil meaning in music. The four processes reflect four ‘levels’—related to realms of reality (Gilgen, 1987) and mental worlds (Pernu, 2017)—at which music acquires referential meaning: cognitive (subconsciously processed), psychological (mental association between referent and music), social (the role of people and situated environments in meaning formation), and cultural (the role of culturally situated habits in forming expectations of the referents of music) (Serafine, 1983; Moisala, 1995; Brower, 2000; Tolbert, 2001; Cross, 2012; Stevens, 2012). We present four processes of framing that more or less respectively correspond to each of these levels, as follows: Fluency, Facilitation, Affiliation, and Fit.

## 3. Framing by Fluency

### 3.1. The Origins of the Framing by Fluency Concept

Our proposed concept of framing by fluency builds on processing fluency theory (PFT). PFT proposes that easier-to-process stimuli are preferred over those that are harder to process (Bornstein & D’Agostino, 1994). The effect has been applied to understanding preferences for aesthetic stimuli such as music (Reber et al., 2004). According to PFT, familiar stimuli are processed more easily than unfamiliar ones, accounting for the common finding (Margulis, 2014; Chmiel & Schubert, 2017) that repetition (a simple technique for increasing familiarity, and therefore more fluent processing) leads to greater pleasure than when the stimulus is unfamiliar (and therefore harder to process). Reber et al. (2004) postulated that individuals attribute the pleasure generated by an easy-to-process stimulus not to the fluency itself, but to characteristics of the stimulus (e.g., its quality) and the experience triggered by the stimulus (e.g., how much it is liked). In other words, a piece that has been previously heard without the awareness of the individual will be preferred more, but the reasoning provided by the listener might (counterintuitively) be to the novelty, rather than the familiarity, of the piece (overviews of misattributions reported in response to music can be found in the following works: Wang & Chang, 2004; Montoya et al., 2017; Mungan et al., 2019).

Applying PFT to the frame itself (rather than the music) makes an unusual prediction about the *form* that the frame takes. If the frame itself is easy to process (e.g., familiar, easy to read, uses high-frequency word/images, looks appealing, is well balanced and proportioned), then, regardless of the content of the frame, the processing fluency it generates will lead to a positive evaluation of the music. Anglada-Tort et al. (2019, Experiment 1) supported this prediction with the finding that music labelled with an easy-to-pronounce title is overall better liked than when the same music is associated with difficult-to-pronounce titles. Simple rhyming of text is also associated with ease of processing, compared to an identical message presented in prose. Empirically, this received support from a study showing that song popularity is aided by rhyming of lyrics (Melvill-Smith et al., 2023). These findings are consistent with the ‘repetition-induced truth effect’, where the information that frames a stimulus repeatedly is more likely to be viewed as truthful and real to a greater extent than equivalent but less frequently repeated framing information (Schnuerch et al., 2021). Thus, while the amount of research on the fluency of the framing material itself has not received much research attention, it may well be that there is a deeply cognitive aspect of framing that affects meaning formation in music, and that it does so quite indirectly.

### 3.2. The Scope of Framing by Fluency

The scope of framing by fluency is overall straightforward because it refers to the form of the frame (e.g., easy to read) and the relationships the individual has with the framing material (e.g., familiarity). To further narrow the scope of framing by fluency, only the fluency of the frame *prior* to its association needs be considered. This is because once the frame provides a context for the music, other factors may obscure underlying effects of the frame’s fluency. To give an example, if material (the frame) is presented about a piece of music each time the music is played, repeated hearings (accompanied each time with the frame) will lead to greater fluency with *both* the music and the frame. Thus, both will become easier to process. It is currently not clear which one would dominate the change in appreciation of the music, though the bulk of research focuses on the music signal, rather than the frame (Chmiel & Schubert, 2017), suggesting that there is an intuition that the frame will contribute less to the effect. If this is the case, then fluency occurring during pairings with the music are less likely to be noticeable, making the impact of fluency at the time of first presentation with the music more important—how familiar it is, how easy and appealing it is to read/hear, and so on.

Another matter concerning the scope of framing by fluency is that information about a well-known piece may be ubiquitous, meaning that the process of ‘fit’ (discussed below) impacts on fluency because of the culturally founded availability of the information which makes it familiar (easier to process). Thus, framing by fluency will be influenced by other processes, but the nature of fluency itself is still essentially cognitive and bottom-up. Framing by fluency can act in a rapid, subliminal manner, which may even be ‘pre-cognitive’ under some circumstances (Kappas, 2006).

## 4. Framing by Facilitation

### 4.1. Positive and Negative Valenced Framing

Facilitation is the process by which particular meaning is highlighted and amplified in the framed material, making it the process that is most directly related to Entman’s (1993) definition. Facilitation is the most researched of the four processes in the Communication subdiscipline, with the main finding being that the perception of an event or object can be emphasised in a positive (or negative) manner purely as a result of the way in which the frame contains linguistic or visual (or any other sensory mode) material which contains positive (or negative) information about the stimulus (Boeynaems et al., 2017; Omondi, 2025; Reynolds et al., 2025). The content of framing by facilitation need only be ‘is’ statements (the music is good, the composer is great…), with ‘because’ clauses being optional and no requirement to be verifiable (e.g., Pickel, 2004). While there are other forms of meaning that can be transmitted by facilitation (such as emotional arousal), we focus this overview on the valence of the framing, reflecting the amount of research interest it has attracted.

Chmiel and Schubert (2020) reviewed evidence for the facilitation process as it applies to music stimuli. The ‘valence’ (positive or negative) of the information matters, with positively framed information being associated with a positive reception to music, in terms of preference and judged quality. Mentioning that a composer was great, describing an impressive curriculum vitae of a performer, or reporting an extraordinary effort required during preparation or performance, can each positively influence the reception of the musician or music to which the frame is referring, showcasing empirically and straightforwardly how meaning in music develops through overt extramusical association.

Even the same recording accompanied by distinctly valenced frames can produce evaluations of the music that are nuanced by each cooccurring frame. That is, if a sound recording of a piece is played while accompanied by positive information of the performance, and then the identical recording is played again, but this time with negative evaluation, the two playings of the same piece are, on average, evaluated differently, and thought to be different performances, influenced by the framed information. The phenomenon is reported by Anglada-Tort and Müllensiefen (2017) as the ‘Repeated Recording Illusion’. In Western music, the title is a ubiquitous form of framing. Positively worded titles can lead to more positive evaluation of the music (Anglada-Tort et al., 2019, Experiment 2).

Labelling the effect as an illusion highlights the surprising impact of framing by facilitation on the quality of a piece of music, something that is (culturally at least) expected to remain stable, particularly in the recorded form, when a digitally recorded piece that is replayed using the same equipment should sound identical upon each playing. As reviewed by Chmiel and Schubert (2020), framing by facilitation comes with a considerable amount of evidence and is perhaps the best understood framing processes.

### 4.2. Negative Framing

The propagation of negative information about a piece of music or musician is a negative frame (reducing facilitation), having an overall negative impact on the evaluation of the music itself. Facilitation can reinforce *or* deride the value of music. It can also impact the emotion evoked by the music, as demonstrated in a study by Vuoskoski and Eerola (2015), who found that music which could normally induce sad feelings could have that emotion intensified when accompanied by framing information that reinforces the sadness of the piece. The valence of the narrative or character in a motion picture accompanied by music also quite likely has an influence on the meaning in the music alone (e.g., see Vuoskoski & Gram-Nilsen, 2023). Evidence is further reinforced in a study by Dasovich-Wilson et al. (2025) which demonstrated long-term impact of music videos on meaning formation of the music.

In an early empirical study, Rigg (1948) showed that, when the description of a piece of music is unfavourable, it is liked less than when the description is favourable. Rigg’s design consisted of three conditions—negative framing (unfavourable comments about the work read by the listener), positive framing (favourable comments), and no framing (no information) provided during listening. These listenings took place a day after initial exposure to the same recordings when no information was presented for all three groups, allowing Rigg to analyse affective responses in comparison to a baseline preference rating. Those who read positively framed information about the pieces reported relatively higher liking scores than those who read negatively framed information. However, the negatively framed condition did not produce negative preference rating scores compared to the baseline prefrence. Rather, on average, they produced positive ratings but to a smaller extent than the positive framing group. We will argue in the Discussion Section that this asymmetry in the valence of the frame is unlikely to be an accident or error in the method applied by Rigg, and we will show how, in putting the four framing processes together, such effects would be expected.

### 4.3. The Scope of Framing by Facilitation

**Figure 1:**
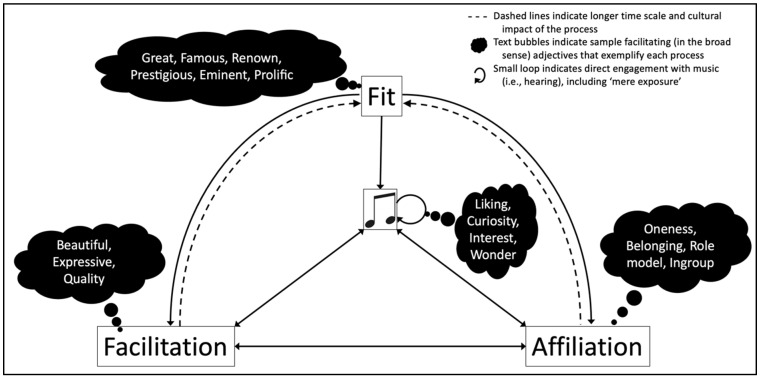
Interactions of meaning formation among music listening (centre) and three framing processes: fit, facilitation, and affiliation.

The facilitation process is at the core of the other framing processes, aside from framing by fluency, because, as far as the above reviewed literature suggests, the valence of its content contagiously transfers to the stimulus itself through no more than suggestion (the valence content of the frame). Framing by fluency indirectly impacts on the experience and evaluation of the stimulus because there is no need for an overtly positive message in the frame. It just needs to be easily (fluently) processed. However, we will see below that the other two framing processes are facilitatory in a broad sense. Affiliation is facilitatory because, overall, it feels good to belong to an ingroup or to identify with a good artist, but it feels bad to be excluded from an ingroup or to find out that an admired artist has acted in a morally despicable way. And we will also see that the process of fit is just a facilitatory process that is acting at a higher (i.e., top-down) level as a result of entrenched personal and cultural habits. We present an illustration of the possible interactions for each of the framing processes in Figure 1.

We therefore distinguish between framing by facilitation in i) a broad sense, where it impacts on affiliation (social) and fit (cultural), and ii) in the narrow sense, where it operates at a psychological level, as, for example, positive messaging of the frame becomes an assumed truth about the music/musician. The process of facilitation acts on the music over a short time frame—the time it takes to reach an evaluation of the music/musician after processing the framing information. That is, as soon as the facilitatory information is presented, it primes (or confirms) the musical experience with that commensurate valence (a positive message about the music primes the music as positive, a negative message primes the music as negative, an emotional message primes the corresponding emotion in the music, and so on). In the narrow sense, facilitation is not a social process, nor is it a cultural process. It is purely psychological.

## 5. Framing by Affiliation

### 5.1. Group Identity

Framing by affiliation builds on the following two extant theoretical phenomena: social identity and parasocial behaviours. In terms of social identity, music can be used as a badge that represents a social affiliation, or a desire to obtain such affiliation (Lonsdale & North, 2009; Lonsdale, 2020). However, there is ample evidence that the effect also works in reverse. Social bonding infuses meaning into the music (Fingerhut et al., 2021). As Spychiger put it: “*context*, and within this a person’s social relationships, play out crucial roles with regard to the nature and quality of musical experiences” (Spychiger, 2017, p. 270, italics from the source).

Social identity theory (Tajfel, 1974) has been applied to musical preferences and taste formation (Hargreaves et al., 2015) by looking at an individual’s use of music as social display or as ‘wearing’ the music as a figurate ‘badge’ of their identity (Frith, 1981, p. 208). These badges can signify individual identity and belonging to a so-called ‘ingroup’ with whom the individual identifies. At the same time the badge can be used to distinguish oneself from a shunned ‘outgroup’ or the ‘other’ (see also Bourdieu, 1987; North & Hargreaves, 2008; Lonsdale & North, 2009; Lamont & Hargreaves, 2019; Lonsdale, 2020; Clark & Lonsdale, 2022). These affiliative frames reinforce the desire to listen to a piece that is identified as part of the individual’s own or group’s identity (e.g., Lawendowski & Besta, 2020).

Subcultural norms and the social desire for bonding are important driving forces in this kind of framing (for a review of various cultural influences on music, see Lamont & Hargreaves, 2021). The effect draws on the principle that Hargreaves (1986) refers as a ‘conformity’ effect. Although the framing is typically social, it can also be a connection with architecture, nature, and so on (Coomber & Harré, 2022), with such connections even triggering musically spiritual experiences (e.g., Matsunobu, 2013).

The use of music for political displays such as protesting (with numersous examples discussed by David, 2013; Friedman, 2013; Martinelli, 2017) also works in reverse. Group identity infuses the music’s meaning particularly when there is a common cause, be it particular social or political (e.g., protest music against injustice), time spent together among friends, experiences of war, and so on. Of the many dozens of published examples, Bradley and Werner (2015) recount numerous uses of popular music by American Vietnam veterans, specifically the role the music played in their group identity and the various meanings the music came to represent.

### 5.2. Social Communities Among Fans

Alongside music acquiring meaning from an available social ingroup, music lovers can engage in parasocial relationships, meaning that they engage in vicarious social relationships with (usually inaccessible) musicians. That is, without necessarily ever meeting musicians, fans engage in one-sided relationships that include watching them, reading about them, fantasising about them, and studying their music, background, lives, and gossip (Giles, 2023). Such engagement is expected of the (music) ‘fan’ who ‘follows’ the musician and their works (A. E. Krause et al., 2018; Kurtin et al., 2019) or, in the more elite guise, the (music) connoisseur, who accumulates knowledge about music and musicians (for a more nuanced discussion of terms such as fandom and connoisseur, see Keller, 2011; Cavicchi, 2014, p. 54). In each of these cases, the music lover acquires knowledge that frames their experience of the music and the musician (Carù & Cova, 2005; Allett, 2012). It may be that the music drives the desire to acquire the knowledge about the musician. However, our perspective focuses on the other side of that coin: the way that socially driven knowledge seeking drives (and usually reinforces) music’s meaning formation through the framing process of affiliation.

Framing by affiliation also takes place through the social circles of the fan or connoisseur, their like-minded friends and acquaintances, or clubs where others also have knowledge or relationships relevant to the same artist(s) (ingroup), while those who do not have such knowledge or interests are excluded or not tolerated (Lamont, 2017; Nummenmaa et al., 2021; Powell et al., 2024). The music those individuals listen to creates an aesthetic attitude different to the aesthetic attitude toward music associated with a fan/connoisseur from an outgroup.

### 5.3. The Scope of Framing by Affiliation

The affiliation process can be interpreted in a broad and narrow sense. In the broad sense, it can include any frame consisting of a social element: for example, even the mere presence of an audience, or the memory of an individual or individuals. Any recommendation encouraging listening to a piece of music (i.e., facilitation) will have some social element, in that the recommendation is likely to have been made by a person, or written by a particular author. Affiliation in the broad sense encapsulates any such social aspect of framing. Music is enjoyed more when ingroup members (such as close friends) are present (Liljeström et al., 2012; Loersch & Arbuckle, 2013). This is also an iteration of facilitation, and has been studied in a variety of disciplines under the banner ‘social facilitation’ (Zajonc, 1965; Brown & Pehrson, 2019). Another example of facilitation and affiliation overlap, again related to ‘social facilitation’, is the positive priming of an enthusiastic crowd at a concert, and the contagious effect this can have on the experience and evaluation of the music (Davidson & Keene, 2019). More than a century ago, Balz (1914) noted the transmission of information about music through social contagion: “[i]n the human herd […] emotions will arise in one as an organic echo of a disturbance in another person” (p. 238). Figure 1 shows how affiliation is acted upon by the facilitation and fit processes, but also encourages listening (centre of Figure 1) and facilitation (e.g., positive valence facilitation for music because it is associated with the ingroup), and how, over longer time periods, it can come to influence fit, because fit is determined in part by influential people (discussed next).

In the narrow sense, affiliation refers to the impact of listening to music as part of the desire to be or remain part of a given social group (e.g., an ingroup, and/or to make a connection with the artist(s)). That is, the music becomes imbued with meanings that made it a badge of identity.

## 6. Framing by Fit

### 6.1. Meaning Acquired Through Enculturation and Cultural Expectation

We borrow the term ‘fit’ from ‘musical fit’, which describes the phenomenon where music can trigger memories and expectations about some knowledge/artefact that was previously associated with the music through habitual pairing (Yeoh & North, 2010a). The origins of the idea stem from classical conditioning theory, where, as a result of previous pairings, the music primes some aspect of the listener’s knowledge (North et al., 2004).

Once established, musical fit can manifest through common association, such as the desire to eat Indian cuisine when Indian music is heard (Yeoh & North, 2010b), or to buy French wine when French music is playing in a liquor retailer (North et al., 1999). In each of these examples, the musical fit is a result of the common connotations that are conjured by the music and the items for sale, such as Indian in the first example and French in the second. That is, when the music–knowledge pairing makes reference to culturally generated congruency, the knowledge is reinforced, whereas an incongruent pairing (i.e., contrary to habits and stereotypes) adds noise to the knowledge (MacInnis & Park, 1991, p. 162; Oakes, 2007).

We apply the term in the context of framing to mean the same relationship between the frame and music, but in the reverse direction. Here, the music (e.g., Indian music) is imbued with meanings that are primed by a previously established match. ‘Framing by fit’ therefore creates an expectation about the music in terms of its previously established referents.

Empirically assessing framing by fit can be found in music research from the mid-20th century. Farnsworth (1950; see also North & Hargreaves, 2008) calculated the amount of space taken up in encyclopedia entries for a number of composers, in order to assess what he referred to as their *prestige*, which can be viewed as a simple measure of framing by facilitation. More space devoted to a particular musician suggests more information that frames that musician, and such entries are usually descriptive but also list the musician’s achievements, which are usually to be lauded. Space devoted to a musician or music in a culturally accessible encyclopedia is an index of prestige, providing a simple quantification of musician/music facilitation.

The process of fit is therefore a form of facilitation that is enshrined at a cultural level, such as through the widespread distribution and availability of artefacts (encyclopedias, biographies, books, articles, reunions, anniversary celebrations, biopics, documentary films, revivals, etc.). This cultural knowledge itself generates expectations of what music will or will not be considered important and worthy of listening. Framing by fit therefore sets up how other frames (facilitatory in particular) are likely to be applied. Fit acts in a top-down manner on all other framing processes (Figure 1, top, centre with downward pointing solid arrows), driving which music and musicians the members of a culture are more likely to select, embrace, and frame through the process of facilitation, in line with the view that culture can act on cognition in a top-down manner (Senzaki et al., 2014).

Anglada-Tort et al. (2019) found that music with the presence of any title was more preferred than music without title. One explanation of this finding is that the verbal title provides a memory cue to the piece, in which case the fluency is the likely process. The framing by fit explanation is that having a title for a piece of music is part of a cultural expectation, a culturally learnt ‘fit’. This explanation can also be used to explain the findings of a study by Margulis (2010), who investigated the effect of different kinds of program notes to accompany music that was familiar and music that was unfamiliar. A difference was noted when presenting factual program notes about the familiar music compared to factual information about the unfamiliar music. The unfamiliar piece was less liked when factual information was presented, in comparison to when the audience member had no program notes at all. Since the program notes were factual and provided by the experimental team, it may have been that the different material in the program notes influenced their facilitation. That is, program notes of an affirmational nature (facilitation) may have helped. But it may also be that when the music is not well represented mentally (i.e., unfamiliar), the listener can self-select the best fit, a phenomenon referred to as ‘self-guided framing’.

### 6.2. Self-Guided Framing as a Way of Generating Fit

Asking individuals to create their own frame while listening to a piece of music can be viewed as a spontaneous indicator of fit. While, logically, the narrative, images, or situations that are brought forth by the listener are triggered by the music (and so not a preconceived frame in the manner in which we have been describing), the phenomenon still provides some insight into framing, and framing by fit in particular. This is because imagery and associations that the listener conjures will be driven by pre-existing norms that are available as mental representations (or schemas/scripts—see Ranta, 2021), easing the listener’s search for the non-music trigger. This is likely to evoke some kind of mnemonic, including stereotypes and other easy-to-process scenarios, that will facilitate the processing of the music itself.

Self-framing was proposed by Chmiel et al. (2022), who assessed the phenomenon empirically. In that study, one condition consisted of participants encountering an unfamiliar piece of music, wherein the listener was encouraged to form mental imagery freely inspired by the music. In this unfamiliar condition, participants produced overall more positive evaluations of the music than the control conditions (implicit exposure and historical information). While idiosyncratic self-guided frames could not be excluded from that study, it would seem plausible that culturally stereotyped narratives would be more likely to be triggered across the population, providing an additional pathway to identify framing by fit. However, the method also conjured triggers that were easy to process, so framing by fit and by fluency were both evident during self-framing tasks. This interpretation is supported by Margulis et al. (2022) who, when examining open-ended responses to pieces of instrumental music, found greater similarity in themes reported by participants who had culturally more in common.

### 6.3. Negatively Valenced Fit

Both positive valenced framing and denigrating a work or creative artist can be found in the historical record. The latter can have a negative impact on aesthetic evaluation (‘negative framing’), but it is not straightforward. In Western art music, elitist attitudes typically belittle notions of popularity, and turn against a composition, creator, or ‘untrained’ listeners when popularity of an elite icon becomes too great. *Wellingtons Sieg oder die Schlacht bei Vittoria*, Op. 91 (1813), one of Beethoven’s admired works during his life time, was noted by Mathew (2006) as “marking the first major defeat of the heroic style […], one that critics have long dismissed as one of Beethoven’s most worthless ‘occasional works’” (p. 21), with Cook (2003) making the observation about the same piece that “the contrast between its popularity and its quality was endlessly reiterated throughout the twentieth century” (p. 3). Thus, when a listener receives negative information about Beethoven’s Wellington’s Victory, the negative assessment of the music could well be primed by the negative fit that has been endorsed by culturally disseminated authority figures.

### 6.4. Fit as Conservative, Automated Processing: Stereotyping and Discrimination

Framing by fit also operates at a more insipid level, drawing on cultural norms that are stereotypes. Shevy (2008) asked participants to assess various aspects of meaning associated with the country and hip-hop genres, showing differences along a number of dimensions concerning the type of performer associated with each genre, with differences that were noted covering the expected ethnicity of the performer through to clothing donned, including specific information about the type of shoes worn. Generally accepted views about music, including the formation of non-conscious emotional stereotyping (Susino & Schubert, 2019, 2020), occur purely as a result of cultural norms. Stereotyping emotion in music refers to the assessment that music is expressive of a particular emotion not because of its musical characteristics, but because of the ‘other’ or exotic cultural association it triggers, such a Japanese Gagaku sounding calm to a listener who is not well versed in that music genre or culture (Susino & Schubert, 2020).

Fit-based framing can occur at an even more sinister level when it surreptitiously leads to the exclusion of groups of people, a common example being the deeply institutionalised stereotypes of Western art music where dead, white males ‘fit’ with the composition of great music in the Western art music canon, implicitly losing sight of fraught opportunities and suppressed achievements of non-white, disabled, and/or non-male gendered people (Strong, 2014; Ewell, 2021; Bombola & Turner, 2022). Thus, framing by fit, when problematised as a politically institutionalised matter, can be changed through progressive, enlightened action directed toward greater diversity and embracing people from perceived or actual discriminated groups. Fit processes, despite their tendency to change slowly, are not necessarily fixed, and can be undone, for example through some morally unacceptable event, such as reprehensible criminal activity, where a once loved creative artist is rapidly forgotten (e.g., Moore, 2022).

### 6.5. The Scope of Framing by Fit

Fit could be viewed as a subcategory of framing by fluency, because, when music is played in an environment that fulfils expectations (fit), its aesthetic experience will be enhanced (Bertinetto, 2024). It is easier to process music when it is framed by fit because cultural forces, such as films, literature, and icons about a musician or their music lead to exposure to those frames, and exposure (in this case, to the framing information) is one of the ways of enhancing fluency. The impact of culture on cognition also reinforces the interaction between these processes (Shore, 1996). That is, fit creates expectations at a cultural level, which therefore impacts what is processed more fluently (namely, expectations, whether transmitted through culture, innate processes or past experiences). Framing by fit is also facilitatory. If an individual grows up hearing that Beethoven is a great composer, that the Beatles revolutionised pop music, that Ravi Shankar opened sitar music to an international audience, that Ella Fitzgerald is an enduring jazz singer, and so on, then those facilitatory pieces of information, disseminated by culture, influence the listener’s perception of the music/performance and the meanings that the music/performers evoke.

Framing by fit overlaps with social factors, for example, because it depends on who exercises control over the amount and type of information that is available for dissemination (consider the role of the editor in the above referred to encyclopedia, or the influencer or disc jockey who decides what pieces should be listened to) (Coase, 1979). With the advent of social media, we also see the increasing role of individuals transmitting frames to other individuals quite rapidly, which was not possible in the pre-digital age, with ‘recommendations’ (‘likes’) and ‘influencing’ acting as framing by facilitation at a directly social level. An analysis by Reisz et al. (2024) showed socially cascaded and reinforced framing (e.g., ‘likes’, or short supportive videos about a song) to be directly attributable to the overall popularity of songs and, thus providing an example of the rapid formation of framing by fit at a subculture level (the subculture that is part of that social media group). In other words, fit can also overlap with framing by affiliation. It can also influence the choice of framing that someone makes at a facilitatory process level, for example, when authoring program notes and even when selecting music pieces for a performance. Consider the most obvious example: if information is not available about a piece of music in one’s culture, it is less likely to be programmed or given airplay in comparison to music that does have information available about it. The same holds for the musician.

While each of the other three processes can therefore be implicated in framing by fit, the unique aspect of the process is that it acts as a top-down (usually culturally formed and stored) driver on these other framing processes, as shown in Figure 1 (see also Colagè & d’Errico, 2020). Arguably, framing by affiliation might be more difficult to dissociate from fit because social contexts are required to do the disseminating, a point to which we will return in the Discussion Section. Framing by fit can also occur at a personal level, such as the triggering of nostalgia and autobiographical memories that also imbue music with meaning. Barrett et al. (2010) showed that this effect is likely to be mediated by personality factors, in particular those prone to nostalgia. Personal fit therefore reflects the learnt (automated) associations that have built musical meaning to individuals that are not limited to large-scale cultural forces, but can (and must) include personal, autobiographical networks.

Thus, the cantus firmus of framing by fit is the establishment of meaning relationships over (usually) long (autobiographical, generational and phylogenetic) time scales, and the fact that they have already been established, ripe for further processing, particularly through fluency and facilitation. Being already present (either as part of one’s culture or personal expectations), framing by fit acts as a driver for framing by facilitation, quite likely influencing how further framing for a piece of music will be fashioned. For this reason, in Figure 1 we show dotted lines from the facilitation and affiliation processes towards the fit process (i.e., they take more time and greater social effort to influence fit), while fit is acting on the other processes (solid line arrows) in a top-down manner, including fluency (which is not shown in the figure).

## 7. Discussion

**Figure 2:**
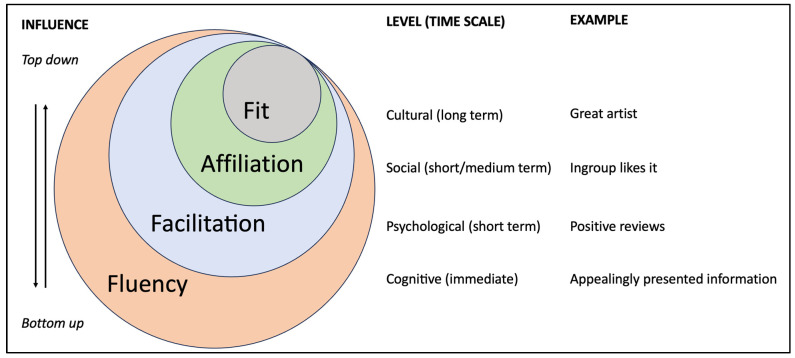
Framing processes mapped onto dependency, influence, and time scale. The four framing processes (fluency, facilitation, affiliation, and fit) are organised according to the top-down (fit) and bottom-up nature of their influence, the level at which they operate (primarily cognitive, psychological, social, or cultural), the time scale over which they tend to operate, and a simple example. Concentric circles indicate which processes are at play during the operation of the other processes. For example, facilitation will take place during the affiliation and fit processes. Affiliation underlies fit because a particular social group (influencers, elites, media, educational institutions) can decide on how fit will be formulated. By contrast, the horizontal stratification (one per process, as shown by the different colour for each process) indicates the narrow sense of how each process operates. See body text for additional examples and further detail. The presentation of the processes is simplified and may change as new evidence emerges; e.g., there is still little evidence for how widespread and reliable the fluency process is, and facilitation is shown as encompassing affiliation and fit, but it may also encompass fluency because high cognitive fluency leads to positive evaluation, which is, therefore, facilitatory.

The four proposed processes of framing work interactively to infuse music with meaning, but each process has a clearly identifiable scope, providing a tool kit to analyse and predict the formation of extramusical meaning. We demonstrated that the framing processes can be distinguished by the rate at which they take effect, with fluency acting on a (potentially) very small time scales, while, at the other extreme, fit can operate over generational and phylogenetic time scales. The processes can also be distinguished as internal processes of cognition (fluency) and psychology (facilitation), through to social (affiliation) and cultural processes (fit). Figure 2 summarises these organisational aspects of the framing processes.

While the processes considerably interact with one another (a suggested set of interactions are shown in Figure 1), fluency can be classified as the most basic (most ‘cognitive’) process because it necessarily acts on every other process at a sensory–perceptual (bottom-up) level. Meanwhile, the process of fit has a more clearly unique processing responsibility, namely the storage of long-term information and the readiness to prime that stored material (top-down). Thus, the process of fit loops back into fluency because the expectations stored in culturally (and personally) formed long-term habits determine what will be processed fluently (cultural expectation leads to greater fluency). This looping is akin to the ‘looping effect’ between culture and cognition proposed by Vogeley and Roepstorff (2009).

The four processes allow for a more nuanced understanding of numerous phenomena that are concerned with meanings triggered in music (referential meaning). To illustrate, we will apply the processes through three case studies taken from the extant literature. The intention is to show how greater clarity can be achieved in the complex matter of musical meaning acquisition, and how the processes can be used to guide further thinking and experimental design.

The case studies were selected to reflect the broad range of questions that the four processes can explain. First, we investigated an early study of music framing by Rigg (1948) to explain why negatively valenced framing—which should lead to negative evaluation through a simplistic (negative) application of facilitation—produces an overall slightly positive valence effect, by considering processes other than framing. We then looked at how the various processes can be applied to clarify unexpected results in a study by Davidson and Keene (2019), where a sound recording is manipulated with non-verbal information. And finally, we applied a historiographic approach to the four ‘effs’ to explain how composers such as Beethoven and Mozart entered and stayed in the musical canon, illustrating an application of the processes beyond music psychology and empirical aesthetics.

### 7.1. Case Study 1: Positive Framing Is More Impactful than Negative Framing

In the study by Rigg (1948), differently valenced facilitatory information (positive, neutral, or negative) was provided to different groups of listeners (see Framing by Affiliation, above). The group receiving the unfavourable program notes had a proportionally lower increase in music liking compared to the favourable and neutral conditions. Thus, although the negative valence frame was associated with lower liking of the music compared to the neutral and positive conditions, the overall liking score was still marginally higher than it was prior to framing. If the process of framing by facilitation is legitimate, and assuming there is no serious concern with the method of the study, one might ask why was the liking not reduced and negative in response to the negative valence framing? This case study examines how processes outside framing by facilitation are implicated in explaining this result.

Previous exposure to music impacts preference, regardless of framing (Hargreaves, 1984; Chmiel & Schubert, 2017). Rigg took a baseline measurement for preference on the day before the framing was provided, so that he could directly examine the extent to which the addition of framing information would impact enjoyment. However, the frame was presented at a separate listening, and so the additional exposure for the listening session that was framed may still have led to increased preference rating. In comparison, Wu et al. (2020) found a considerably stronger effect of liking as a result of exposure than any of four framing conditions—one of the few studies to test both referential (via framing) and absolutist (via exposure) meaning in the same design. The power of simple musical exposure on affective response should not be underestimated (Margulis, 2014; Chmiel & Schubert, 2017). If music is listened to, despite a negative message, the mere exposure to the music may still be acting upon the evaluation of the music, possibly inhibiting the impact of negative valenced framing. As Zajonc (2001) noted, “positive affect can be attached to a stimulus by virtue of mere repeated exposures” (p. 226).

An alternate explanation is that the framing material itself may have become increasingly fluent to process as the experiment unfolded on the second day. This is because the messages about each piece in the negative framing (unfavourable) condition concerned German themes, and so repeatedly reading that information from one stimulus to the next may have increased its processing *fluency*, thereby impacting positively on the preference for the piece at a subliminal level. We should also note that there may have been some ambiguity in some unfavourable condition descriptions. For example, one of the four negatively framed pieces was by Beethoven, in which the accompanying notes of the negative valence condition merely mentioned that he was a German composer, even pointing out that this was more than a century before Germany became an Imperial state.

The other three negatively framed pieces were by Wagner, and for two of these pieces reference was made to Hitler’s adoration of Wagner and his music, and the embracing of his music for patriotic, nationalistic purposes. Thus, the negative framing used *affiliation* to try to turn the listener against the piece. The paper was published not long after World War II, and data were probably collected before the end of the war given the present tense pronouns for Hitler in the descriptions read by the participants. The participants were students from the USA (the ingroup in this case), and so we can surmise that they were likely to seek avoiding affiliation with the leader of an undesirable group—Nazis (the outgroup, who their country was at war with at the time). The use of the same negative (affiliation) information may have increased the fluency of the message. In the third of the three unfavourably framed Wagner pieces, it was the composer’s own morality that was questioned, referring to his involvement in “an affair which resulted in a divorce” (Rigg, 1948, p. 79), possibly contributing to a poor evaluation due to negatively valenced framing by *affiliation*. The negative valenced frames were ambiguous with regard to *facilitation*, without any outright negativity about the quality of the music. The refusal of Rigg to refer negatively to the music itself or the skill of the composer reflects the realistic view that the listener may have already been familiar with Wagner’s (positive) reputation as a composer through cultural norms through framing by fit. Thus, in our reanalysis of the data, negative valence could only be achieved through a process of (negative) affiliation.

For this particular case study, our reanalysis of Rigg’s study in terms of the four framing processes and the absolutist process of mere exposure suggests that the process of mere exposure to the music, plus framing by *fit* and a possibly small contribution of framing by fluency, were acting against the intended negative impact of the framing by negative affiliation to produce an overall partial cancellation of the intended negative framing. This case study shows how the framing processes of fit, facilitation, fluency, and affiliation can be mobilised to explain musical response, but still be insufficient to fully explain results, with exposure to the music itself being considered as part of meaning formation (Stalinski & Schellenberg, 2013).

### 7.2. Case Study 2: Crowd Sounds Manipulated on a Sound Recording

Davidson and Keene (2019) examined listener reception to music recordings with and without digitally edited-in crowd noises. We suggest that framing by *facilitation* was expected when crowd noise is edited-in because positive cheering is expected to cue listeners to the positive nature of the music. Crowd sounds that are more harmonious (as distinct from dissonant ones) would also serve to enhance the aesthetic experience because of improved processing *fluency* of the framed information: harmonious crowd sounds are processed more fluently than dissonant sounds.

Despite this, the researchers found that the effect of the edited-in crowd noise was weak and argued that it would have been stronger had the crowd noises taken place during a live concert, rather than edited onto a studio recording. That is, the crowd effect should work better if the environment produced a better *fit* (live music where crowd sounds are culturally expected). And, if the music used in the recording was by a musician that the listener followed (was a fan), this would further strengthen the aesthetic response, through framing by *affiliation*, with the listener finding in the music a sense of belonging which psychically merges with a sense of bonding and oneness with those who are part of that audience. Thus, any or all of these framing processes may be working together and overlapping to different degrees, to influence meaning formation and maintenance.

### 7.3. Case Study 3: Entry into the Canon: Beethoven and Mozart

DeNora (DeNora & Mehan, 1994; DeNora, 1995) describes in detail the cultural circumstances during and after Beethoven’s lifetime that led him to become an integral cultural figure, with numerous examples of *facilitation* including adoring, influential followers such as E.T.A. Hoffman, Arthur Schopenhauer, and Robert Schumann. Once Beethoven became an accepted cultural icon representing genius, innovation, and greatness—as a result of persistent positive facilitation by his followers (*affiliation*)—the ‘universal’ acceptance of Beethoven’s greatness became the cultural norm. This norm is reflected and reiterated in encyclopedias and the media, and the interest it generates is passed on generationally, leading to a continuing tradition of followers, some of whom themselves will write books analysing and adoring the composer, to which another generation is exposed. Framing by *fit* therefore informs how other framing processes operate, such as framing material for program notes (*facilitation*), as well as the absolutist meaning formed by further exposure because of the widespread visibility of music. Framing by fit can therefore reinforce itself over long time periods.

The framing of Beethoven by fit was well established by the middle of the 19th century for Western European middle class and elite society, with regular reinforcement of Beethoven’s greatness, including narratives of who would be able to match or surpass him—Wagner and Brahms being two of the several famous examples (Bonds, 1992; Pederson, 1993).

This case study shows how facilitative framing of Beethoven’s music during and immediately after his lifetime by his followers (those seeking to be affiliated with Beethoven, and also wielding influence on others through entrepreneurship, journal editing, etc.) would shift these processes to framing by fit, where the association between Beethoven and his ‘greatness’ spread through the subcultures where the frames were disseminated (Figure 1). Framing Beethoven is a rather unusual case that would impact on the framing of Western art music as an elite art form, meaning that, to gain access to those elite circles, the framing of affiliation is at play, that one would seek out and enjoy such music because of these prospective social affiliations. That is, framing by fit would encourage and lead to further framing by affiliation in subsequent generations, with the music and the myth of Beethoven presented through framing by facilitation. ‘Beethoven is a great composer’ is a message that itself, consequently, becomes a ubiquitous statement in Western elite culture, the ubiquity making the phrase *fluent* to process.

Beethoven and his greatness as a composer is therefore an example of the *fit* process operating at a long-term, culturally vindicated scale that is still widespread today (Farnsworth, 1958; Ceulemans, 2010; Gómez-Vega & Herrero-Prieto, 2019). This relationship established the fit for the composer—the sort of expectations that are generated when listening to music. Some musicologists have used this fame and parallel notions of quality to question parts of Beethoven’s repertoire, possibly driven by works that slip into popularity (an ‘outgroup’ for the elite musicologist, as we have summarised above; see the reference to Wellington’s Victory under Framing by Fit).

Mozart, like Beethoven, is another pinnacle of Western art music. An analysis of reviews of a piece of music (Spitzer, 1987) found a marked difference in assessments depending on who the reviewer thought the composer was. Reviews of the work, when thought to be by Mozart, were mostly positive, but, when the identity of the composer of the same work was brought into question, the judged quality diminished notably. Spitzer asserted “the authorship—or attribution—of a musical work may influence both the critic’s experience of that work and the critic’s judgment” (p. 320).

In his analysis, Spitzer tabulated the various descriptions of the reports made depending on the different identities of the composer, with the ‘authentic’ Mozart attribution producing gushing praise, in contrast to the work thought to be by a ‘spurious’ composer, which were considerably more disparaging. When the work is by Mozart, a positive framing by fit process is in action, and, by this example, influencing both highly knowledgeable and naïve listeners.

Music by (or thought to be by) the ‘great’ composer is evaluated positively through framing by *fit* (music by Mozart ‘fits with high quality’ is a culturally established belief). Individuals will have their views of Mozart reinforced by the positive reviews (and diminished when the composition is by an unremarkable composer), impacting on framing by *facilitation*. Those who wish to be seen as experts, connoisseurs, fans, or knowledgeable music appreciators with a desire to be part of or to be accepted into the culture that the reviewers in Spitzer’s (1987) study represent, are provided with an *affiliative* frame by allowing themselves to be tilted by the sentiment of the information from these desirable or actual ingroup sources. This finding was replicated to some extent with a more quantitative design, where participants covering broad age spans rated music framed as being by Mozart or the lesser known composer Mysliveček (Fischinger et al., 2020). The study only replicated the framing by *fit* effect (that, if it is framed as Mozart, the same piece of music will be better—more liked and perceived as having a higher “quality of artistic expressivity”) as an interaction with age, and to some extent by musical expertise. The younger and musically more naive participants were more overall influenced by fit (Mozart compositions were rated higher) than older or more musically experienced participants. Thus, there is still work to be carried out in understanding the process and influence of musical fit.

## 8. Conclusions

In this paper we have applied framing as a basis for understanding the (referential) processes through which meaning (including pleasure) imbues music. We identified evidence for four framing processes. The most overtly influential process of framing is by fit, where norms and regularities exist in culture or in a well-established, automated set of personal experiences. Framing by fit influences what the individual is exposed to because it drives cultural and habitual dissemination of music that has developed over a long period of time. It therefore acts in a top-down manner and is able to exert influence on the other three processes discussed. Affiliation is the role of social and environmental connection in musical meaning. An individual who has not experienced a piece of music as a result of exposure by fit may find another pathway for exposure to music: for example, via a friend (Reisz et al., 2024). We suggest that the social connection—whether a friendship, or desire to join or maintain any ingroup allegiance—provides another pathway, not just for the kind of music that will be heard, but to the meaning of the music. That is, the social setting, be it connections with others or parasocial relationships with the musicians, frames musical meaning. This non-verbal framing can also be through contexts that are not directly social, such as framing musical meanings through a connection with nature. Framing by facilitation is the most directly related to traditional definitions of framing, where the content of the framed information directly encourages or deters listening. The valence (positive or negative)—but also other aspects (such as the emotions)—of the content is applied to the music through the psychological principle of suggestibility, a fundamental aspect of framing, but also through contagion. Another process we identified was fluency, which acts at a cognitive, potentially subconscious level, over very short time scales, and in a bottom-up manner, and is based on the form (not the content) of the frame or the relationship the perceiver has with the frame (e.g., familiarity), regardless of the content.

Numerous labels could have been used to label the processes (Table 1). Our choice to use labels that are alliterative using the ‘eff’ phoneme reflect an application of processing fluency itself, to make the processes easier to recall. Table 1 also shows selected terms that demonstrate examples of overlapping labels (italicised items).

We argue that the four processes show potential to explain how musical meaning comes to be in a more systematic and focused way than was possible before. This includes the much-researched area of musical preference, with all four processes showing how music can come to be loved (or hated) as a result of the extramusical connections that are formed through framing. We also demonstrate how framing can explain the results of existing empirical studies and historical findings by tracing historical events through the lens of the four ‘effs’ and analysing how those events make their way, via framing by fit, into the future, why a piece is judged differently if the name of the composer is changed, and why it is that negative framing does not produce a straightforward dislike of the negatively framed music (because manipulating one process only might not be sufficient). The case studies exemplified the many possible applications of the framing processes that could be applied in future research and so promise to lead to a better understanding of musical experience. The four ‘effs’ hold promise in better understanding several other phenomena in music, such as the role of lyrics in contributing to meaning in music, which, instead of being treated as a separable channel from the music (Stratton & Zalanowski, 1994; Ballard et al., 1999; North et al., 2021), could be one or more framing processes wrapping around the music and its meaning.

Nevertheless, there are some caveats. The amount of evidence for the framing processes in music meaning formation is convincing but not definitive. We mentioned that framing by fluency has the least empirical evidence, and some may even dispute that ease of processing is a necessary component of art experience and meaning formation (Robinson, 2020, p. 205). Issues such as habituation of (Harrell et al., 2021, p. 980; Stanulewicz-Buckley & Cartwright, 2025, p. 24) and irony used in (Clift, 1999; Burgers et al., 2016) the framing material (e.g., through overuse, or culturally obvious incorrect use) require attention in future research. As cited by Fischinger et al. (2020, p. 581), the German philosopher Albrecht Wellmer considered that verbally presented framing may even distract the listener from the musical experience itself. The intention of this paper is, therefore, to act as a starting point to a potentially fruitful paradigm for understanding meaning in music. Above all, we hope to present a theoretical framework that can help to more clearly and systematically focus research on meaning formation in music.

## Figures and Tables

**Table 1:** Brief definition and terms that describe, or are alternate labels (similes), for each framing process.

Framing Process Label	Fluency	Facilitation	Affiliation	Fit
Nature of the framing process: brief definition	Cognitive: form of and relationship with frame	Psychological: evaluation in the frame content	Social: social (and environmental) content	Cultural: historically primed information
Sample descriptions and alternate labels	Aesthetic *appeal* ^1^ Balance Ease of processing Cognitive *expectation* ^1^ Familiarity Simplicity	*Appeal* ^1^ (general) *Eminence* ^1^ *Greatness* ^1^ Positive reinforcement Positive valence *Prestige* ^1^ Suggestibility Support Valence contagion	Belonging Community Conformity Identity Ingroup connection Oneness Parasocial connection Social display/badge Social inclusion/exclusion Social learning	*Eminence*^1^ Enculturation *Greatness*^1^ *Prestige*^1^ Stereotyping Cultural norm/*expectation* ^1^ Habits

^1^ Words shown in *italics* appear in more than one column when the process is applied in the broad sense (see Figure 2 and main text for narrow and broad scope of the four processes).

## Data Availability

Data is contained within the article.
